# Gallstone extraction from a back abscess resulting from spilled gallstones during laparoscopic cholecystectomy: a case report

**DOI:** 10.1093/jscr/rjab293

**Published:** 2021-07-23

**Authors:** Saqib Mehmood, Sohail Singh, Chukwuemeka Igwe, Chekwas O Obasi, Rhys L Thomas

**Affiliations:** General Surgery, Croydon University Hospital, London, CR77YE, UK; General Surgery, Croydon University Hospital, London, CR77YE, UK; Internal Medicine, King Mill Hospital, Sutton-in-Ashfield NG17 4JL, UK; General Surgery, Croydon University Hospital, London, CR77YE, UK; General Surgery, Croydon University Hospital, London, CR77YE, UK

## Abstract

Laparoscopic cholecystectomy is a routinely performed surgery nowadays. However, it is associated with certain complications. Gall bladder perforation during the procedure can result in spilled and lost gallstones. Lost gallstones most commonly cause intra-abdominal infection. However, very rarely, they can be associated with troublesome retroperitoneal abscess formation. We present a case where a lost gallstone caused a retroperitoneal abscess formation and was retrieved from a back abscess in the right paraspinal region.

## INTRODUCTION

It is estimated that 15% of the adult population suffers from cholelithiasis. While the majority of patients are asymptomatic, those who are symptomatic with cholelithiasis can be offered a laparoscopic cholecystectomy [1, 2]. Laparoscopic cholecystectomy is the preferred approach over open cholecystectomy due to its association with reduced length of hospital stay and shorter recovery time. It is one of the most routinely performed elective operations [3].

Although laparoscopic cholecystectomy is associated with a better outcome, gallbladder perforation and subsequent stone spillage occur more frequently in laparoscopic cholecystectomy than open cholecystectomy, with the incidence ranging between 8 and 30% of all procedures [3–5]. Intraperitoneal abscess formation is an important and recognized complication of dropped gallstones. However, very rarely spilled gallstones may migrate into retroperitoneum, causing retroperitoneal abscess formation [6].

Here, we present a case study where a patient suffered from retroperitoneal abscess formation due to a spilled and lost gallstone a few years after undergoing laparoscopic cholecystectomy. As a result, the patient suffered from a recurrent back abscess in the right paraspinal region until he underwent incision and drainage, and gallstone was retrieved from the abscess cavity.

## CASE PRESENTATION

**Figure 1:**
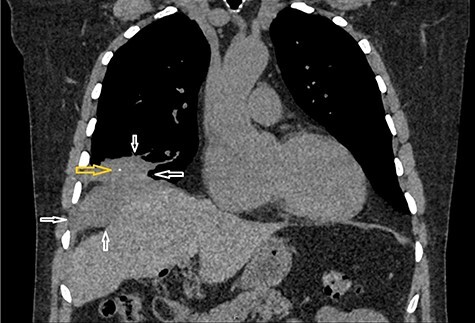
CT scan chest coronal view: Figure demonstrates a mass lesion at the right lung base with an area of calcification. White arrows demonstrate the edges of the mass lesion. Yellow arrow denotes the area of calcification in the mass.

A 65-year-old gentleman with no comorbidities underwent elective laparoscopic cholecystectomy for symptomatic gallstones in 2013. His operation was complicated by intra-operative spillage of gallstones, which were retrieved. The patient remained well until 2017 when he developed a long-standing dry cough. Computed tomography (CT) scan chest showed a right lung base mass lesion with a large area of contact with diaphragmatic pleura, also abutting the right lobe of the liver. A small area of calcification was seen in the mass lesion thought to be part of the malignant process (Fig. 1).

**Figure 2:**
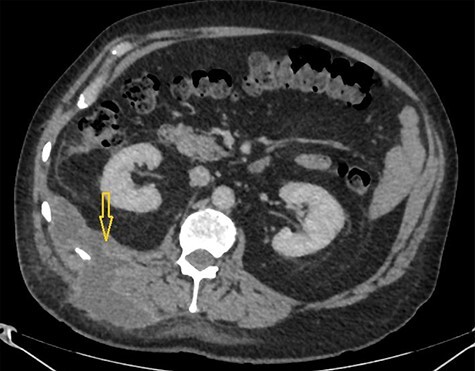
CT scan abdomen demonstrates a retroperitoneal abscess tracking through an intramuscular opening onto skin in the right paraspinal region.

Subsequent positron emission tomography scan and right pleural biopsies only showed inflammatory changes, and no evidence of malignancy was found. This mass lesion was followed up with a CT scan at 6 months interval that showed complete resolution.

Two years later, the patient developed fever and painful swelling over the back in the right paraspinal region. CT scan abdomen showed a large abscess in the right paraspinal region with no apparent cause demonstrated. This abscess was drained with a pigtail drain under ultrasound guidance.

A year later, the abscess recurred, and the patient required acute hospital admission. Inflammatory markers were observed to be elevated, C-reactive protein of 125 mg/L. His liver function test remained unremarkable. Abdomen was soft but tender in right flank.

A CT scan abdomen was performed, which demonstrated a large retroperitoneal abscess behind the liver tracking through a small intramuscular opening onto the skin over the upper aspect of the back (Fig. 2).

**Figure 3:**
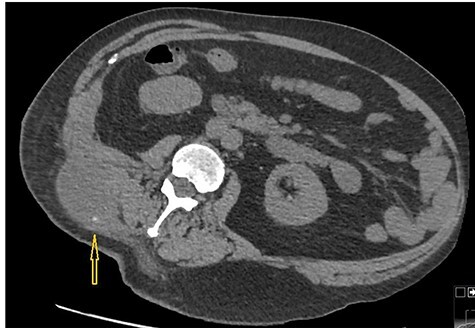
Non-contrast CT abdomen: CT scan demonstrates a small hyper dense calculus in the abscess marked by an arrow. Here patient is lying in slightly left lateral position due to pain caused by the abscess.

Because of previously identified calcification in the mass lesion and history of laparoscopic cholecystectomy, a non-contrast CT scan abdomen was obtained to ascertain if spilled gallstones were responsible for the recurrent abscess formation (Fig. 3).

**Figure 4:**
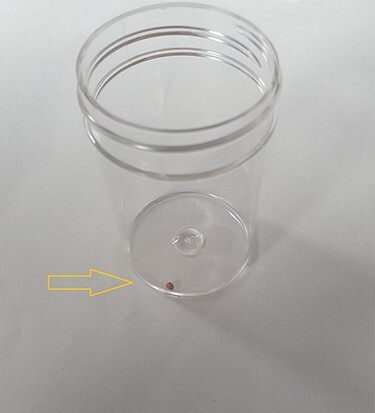
Image shows a small calculus removed during incision and drainage of the back abscess.

Incision and drainage of the back abscess were performed under general anaesthesia. Almost 200 mL of frank pus were drained, and after a washout, a corrugated drain was left in place. Also, during the procedure, a small gallstone was identified and removed (Fig. 4).

Since the operation, the patient has done very well and is symptoms free to date.

## DISCUSSION

Gallbladder perforation is a relatively common complication of laparoscopic cholecystectomy, reported as high as 40% in some studies, resulting in bile and gallstone spillage [3–5]. In most cases, gallstone and bile spillage will not result in any further harm to the patient. On the other hand, it may induce an inflammatory response resulting in intra-abdominal abscess formation. One review estimates complications due to lost gallstones occur approximately in 1.7 per 1000 laparoscopic cholecystectomies [7].

A spectrum of clinical presentations can arise due to a spilled gallstone, varied by many factors, including patient comorbidities, type, size and the number of spilled gallstones [8]. The patient can present within a year to multiple years postoperatively with vague or localized abdominal pain, a mass, fistulas, fever or general malaise [8]. The most common clinical manifestation of spilled gallstones is an intra-peritoneal abscess formation, followed by subphrenic abscesses [8, 9]. However, the least common site of abscess formation is retroperitoneum, as seen in our patient [9].

Spilled gallstones are treated as a foreign object by the body, and as a result, an inflammatory response ensues, which may result in local fibrosis around the stone, sometimes with partial reabsorption. However, spilled gallstones can also erode into the adjacent peritoneum resulting in extraperitoneal inflammatory response leading to abscess formation, as happened in our case [10, 11].

There is currently insufficient evidence on whether a laparoscopic cholecystectomy should be converted into an open cholecystectomy if gallstones are lost in the abdomen. While spilled gallstones are removed with greater ease in an open cholecystectomy by irrigation, the risks of switching to open cholecystectomy and inherently low rates of further complication due to spilled gallstones itself have to be weighed up in individual cases [7].

With the benefit of hindsight, mass-like changes at the right lung base were subsequently thought to be the subphrenic inflammatory response as a result of lost gallstone. The area of calcification in the mass was thought to be the spilled gallstone, which later migrated and was retrieved from the abscess cavity. This mass lesion had initially led to a cascade of cross-sectional imaging investigations and invasive testing, adding to the morbidity of the patient.

We conclude that high index of suspicion for spilled gallstones is required when investigating recurrent intraperitoneal or retroperitoneal abscess formation in an individual with a surgical history of cholecystectomy. We also want to emphasize that management in such cases should involve the removal of the gallstone in addition to intravenous antibiotics and drainage of the abscess; otherwise, the abscess may recur quickly.
